# Opening a Can of Worm(‐like Micelle)s: The Effect of Temperature of Solutions of Functionalized Dipeptides

**DOI:** 10.1002/anie.201705604

**Published:** 2017-07-28

**Authors:** Emily R. Draper, Hao Su, Christopher Brasnett, Robert J. Poole, Sarah Rogers, Honggang Cui, Annela Seddon, Dave J. Adams

**Affiliations:** ^1^ School of Chemistry University of Glasgow Glasgow G12 8QQ UK; ^2^ Department of Chemical and Biomolecular Engineering Whiting School of Engineering Johns Hopkins University 3400 North Charles Street Baltimore MD 21218 USA; ^3^ School of Physics HH Wills Physics Laboratory University of Bristol Tyndall Avenue Bristol BS8 1TL UK; ^4^ School of Engineering University of Liverpool Liverpool L69 3GH UK; ^5^ ISIS Pulsed Neutron Source Rutherford Appleton Laboratory Didcot OX11 0QX UK; ^6^ Bristol Centre for Functional Nanomaterials HH Wills Physics Laboratory University of Bristol Tyndall Avenue Bristol BS8 1TL UK

**Keywords:** dipeptides, gels, low-molecular-weight gelators, micelles, rheology

## Abstract

A simple heat/cool cycle can be used to significantly affect the properties of a solution of a low‐molecular‐weight gelator at high pH. The viscosity and extensional viscosity are increased markedly, leading to materials with very different properties than when the native solution is used.

The process by which a gel is formed using a self‐assembled low‐molecular‐weight gelator (LMWG) is critical in determining the final properties of the gel.^1^ There are many reports attempting to link the molecular structure of the gelator to the gel properties; however, it is possible to produce gels with very different properties from a single gelator by varying the process.^2^ Since predicting new gelators from first principles is still difficult,^3^ it is extremely useful to know that many properties, such as the stiffness, recoverability, and opacity can all be varied significantly using a single, robust gelator.

Gel formation requires assembly into one dimensional structures, followed by the entanglement and/or cross‐linking of these structures.^4^ Hence, it is common for this process to be kinetically driven and the method of gelation has a significant effect on the outcome.1a A further complication is that the gelator must first be added to the solvent pre‐assembly. For organic solvents, it is common to suspend the insoluble gelator in the solvent, and heat to dissolve the gelator; cooling then leads to assembly. In water, this process can also sometimes be achieved, but it is more usual to utilize a trigger, such as a change in pH, to go from a “dissolved” gelator to the gel.^5^ In some cases, the “dissolved” gelator is in fact dispersed as a surfactant‐like aggregate.^6^ The transition from this aggregate to gel fibers is largely process‐controlled and therefore very unlikely to reach a thermodynamic minimum.

Functionalized dipeptides are highly effective hydrogelators.^7^ Dipeptides functionalized at the *N*‐terminus with a naphthalene are robust and effective gelators.^8^ The free *C*‐terminus allows dispersion in water, and a trigger for gelation. At high pH, these can be dispersed by adding base to deprotonate the free carboxylic acid. Typically, spherical or worm‐like micelles are formed,6b although this is concentration dependent.^9^ We have described this scenario in detail and have shown that solutions of the worm‐like micelles can be gelled at high pH by adding a divalent cation.^6^ Herein, we show that the properties of the solutions and resulting gels are strongly affected by a simple pre‐heating step. This effect has important implications for these gelators.

Solutions of 2NapFF (2NapFF=2‐(naphthalen‐2‐yl)acetamido)‐3‐phenylpropanamido)‐3‐phenylpropanoic acid; Figure 1 a) were prepared at a concentration of 10 mg mL^−1^ by adding sodium hydroxide to the dipeptide in water. After stirring overnight, a slightly viscous solution was formed at a pH of approximately 11. We have previously shown that these solutions contain worm‐like micelles.6b, 9 These solutions are susceptible to shear forces, exhibiting enhanced extensional viscosity after exposure to shear rates of between 10^3^ and 10^6^ s^−1^.^10^ This effect was weak and difficult to reproduce exactly.

**Figure 1:**
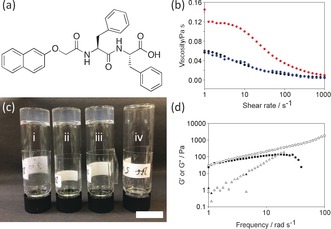
a) Structure of 2NapFF. b) Viscosity of a solution of 2NapFF (10 mg mL^−1^; pH 11) at 25 °C (•), heated to 40 °C for 2 min (•), and recooled to 25 °C (•). c) Photograph of a solution of 2NapFF (10 mg mL^−1^; pH 11) heated to i) 70 °C, ii) 60 °C, and iii) 40 °C, and subsequently cooled; iv) no heating (scale bar=2 cm; photographs taken 3 h after recooling). d) Frequency sweep of a solution of 2NapFF (10 mg mL^−1^; pH 11) before a heat/cool cycle (*G*′ (▪); *G*′′ (□)) and after a heat/cool cycle (*G*′ (▴); *G*′′ (▵)). Note, much of the *G*′ data cannot be plotted as it is in the noise and too small for the instrument to detect. All data were collected at 25 °C.

Herein, we report that heating to elevated temperatures does not change the viscosity of the solutions (Figure 1 b). On recooling, the samples became significantly more viscous (Figure 1 b), and formed materials that were self‐supporting for at least 8 h (Figure 1 c). Overnight, the samples fell to the bottom of the tube. Very similar materials were formed if the heating was between 40 and 70 °C, and samples that could be inverted were also formed after cooling from a short period of heating with a hot air gun. Whilst the samples become self‐supporting, it is clear from the rheological data that these are not true gels. The storage modulus (*G*′) does not dominate over the loss modulus (*G*′′) by one order of magnitude; rather the values are very close, indicating a more elastic type of material (Figure 1 d), and there is some frequency dependence. The *G*′′ for the two solutions also highlights the significant difference in the viscosity of the two solutions near the rest state. This zero‐shear viscosity can be determined by the slope of *G*′′ versus *ω* in the limit of vanishing frequency or, as this terminal regime is not approached for the heat/cool cycle sample, roughly estimated with *G*′′/*ω* at *ω*=1 rad s^−1^.^11^ Before heating, $\frac{G''}{\omega }$ ≈0.5 Pa s, whereas after the heat/cool cycle $\frac{G''}{\omega }$ ≈50 Pa s; that is, two orders of magnitude higher. All of this is clearly important as it is common for tube inversion to be shown as the only test of gelation.^12^

The increase in viscosity was observed over a range of concentrations (Supporting Information, Figure S1), with materials that could be inverted forming at concentrations as low as 5 mg mL^−1^ (solutions at 3 and 4 mg mL^−1^ were visibly more viscous after a heat/cool cycle, but did not support inversion). The concentration range coincides with our previous work on the phase diagram for 2NapFF, where we showed that worm‐like micelles are formed above around 1 mg mL^−1^, with a significant increase in the viscosity above 5 mg mL^−1^.^9^ Hence, this heat‐induced behavior requires that specific aggregates exist at room temperature, rather than this being a heat‐induced structural transition.

The pH is a critical parameter; at 10 mg mL^−1^, samples that could be inverted were prepared after a heat/cool cycle as long as the pH was above 9.48 (Supporting Information, Figure S2). Typically, for this kind of molecule, the apparent p*K*_a_ of the carboxylic acid is around 6,^13^ and can be concentration dependent. At 10 mg mL^−1^, titration of the 2NapFF shows an apparent p*K*_a_ at around 8.5 (Supporting Information, Figure S3). After a heat/cool cycle, the p*K*_a_ slightly increased to around 9.0, but it should be stressed that the high viscosity of this solution made this measurement difficult. We note that elsewhere heat/cool cycles have been used during a pH titration to establish the p*K*_a_ and it is possible that this leads to errors on the basis of the current work. Nonetheless, for the viscosity increase to be observed, the pH needs to be above the apparent p*K*_a_ of the 2NapFF, implying that significant charge is required on the worm‐like micelles.

The samples exhibited significant extensional viscosity after heating and cooling, becoming very “stringy” (Supporting Information, Figure S4). We therefore undertook experiments to quantify the resistance of the solutions to extensional/elongational deformations using a capillary break‐up extensional rheometer (“CaBER”).^14^ In this device, a liquid bridge (2 mm in length) is formed between two circular discs 4 mm in diameter, which are then rapidly pulled apart (ca. 50 ms). The resulting unstable fluid filament consequently thins down under the action of surface tension until finally breaking. The diameter of the filament (D) is observed as a function of time (*t*) using the equipment's laser micrometer (resolution ca. 10 microns). Although the filament diameter data can be post‐processed into an (apparent) extensional viscosity, the standard method to quantify extensional effects^15^ is by an exponential fit to the filament diameter as a function of time in the elasto‐capillary regime to determine a characteristic relaxation time (*λ*; more correctly, a characteristic time for extensional stress growth). Representative plots are shown in Figure 2 a where the effect of the heat/cool cycle can be seen to increase this characteristic time *λ* by almost two orders of magnitude, from 25 ms before heating to a relaxation time of 1.35 s after a heat/cool cycle.

**Figure 2:**
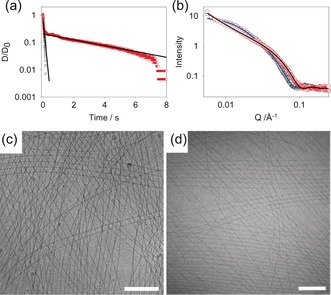
a) Diameter–time data from CaBER experiments, including exponential fits to obtain estimates of relaxation time (*λ*). Key: a freshly prepared solution (10 mg mL^−1^; pH 11; (○)), a sample after heating and cooling (○), exponential fits (—). b) SAXS data (intensity vs. scattering vector (Q)) for a solution of 2NapFF (10 mg mL^−1^; pH 11) before heating ((○); fit (—)) and after heating and cooling ((○); fit (—)). c,d) Cryo‐TEM of a solution of 2NapFF before heating (c) and after a heat/cool cycle (d); scale bar=200 nm.

To explain this behavior, we used small‐angle X‐ray scattering (SAXS). Before heating, the scattering data fitted best to a flexible cylinder with a radius of 40.5±0.2 Å, a Kuhn length of 63.0±2.4 Å and a length of 949.6±5.6 Å (Figure 2 b; Supporting Information, Figure S5). After a heat/cool cycle, the scattering data still fitted best to the same model, but with a radius of 34.0±0.1 Å, a Kuhn length of 339.7±3.4 Å and a length of 4976.4±232.8 Å (Figure 2 b; Supporting Information, Figure S5). In both cases, the absolute length is beyond the resolution of the fit, but it is clear that the length increases after the heat/cool cycle, as does the Kuhn length, showing that the flexibility decreases. The radius decreases after the heat/cool cycle. Small‐angle neutron scattering (SANS) data was also collected (Supporting Information, Figure S6 and Table S2). In agreement with our previous work,^9^ the data before heating is best fit to a hollow cylinder with a radius of 36.9±0.1 Å and a core radius of 19.1±0.2 Å. After a heat/cool cycle, the radius decreases to 31.2±0.1 Å (in line with the SAXS data) and the core radius also decreases to 9.2±0.3 Å. These data lead us to suggest that the 2NapFF assembles into a coiled structure with a central core (Figure 3 a); the heat/cool cycle leads to partial dehydration of the core and an extension in the length. The cryo‐transmission electron microscopy (cryo‐TEM) images of the solution before and after heating (Figure 2 c and d, respectively; Supporting Information, Figures S7 and S8), show that both solutions contain long anisotropic structures with similar diameters (Supporting Information, Figure S9).

**Figure 3:**
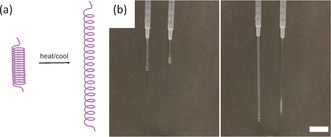
a) Illustration showing how dehydration of the core of the hollow tube could lead to extension of the length. b) Stills from a video showing solutions of 2NapFF (10 mg mL^−1^; pH 11) being pushed out of a syringe. Left, after ca. 0.3 mL has been pressed out; right, after ca. 0.7 mL has been pushed out. In both cases, the solution on the left has been exposed to a heat/cool cycle and the solution on the right has not. Scale bar=2 cm.

Further insight into the heat/cool cycle comes from fluorescence, infrared (IR), and nuclear magnetic resonance (NMR) spectroscopy. Immediately after heating, fluorescence is significantly quenched as compared to the initial solution (see fluorescence spectra in the Supporting Information, Figure S10). There is no red‐shift in the peak, which implies that there is no significant change in the local packing. On cooling, the intensity increases again, but does not reach the original value. Normalization of the data shows that there is a slight increase in the tail of the fluorescence, although the position of the main peak has not changed significantly. These data therefore correlate with a similar molecular packing of the naphthalene rings. The IR data (Supporting Information, Figure S11) shows relatively little change before and after heating, with the main difference being the shift of a peak from 1647 cm^−1^ to 1654 cm^−1^ on heating; this peak is then lost on cooling. This data implies a subtle change in the packing after a heat/cool cycle. In the NMR spectra, peaks are initially broad and integrate at a lower intensity than might be expected (Supporting Information, Figure S12); this is due to the presence of the worm‐like micelles, as discussed previously. On heating, the peaks become better resolved and the integral of the aforementioned peaks increases with respect to the internal standard. This implies that the 2NapFF is more soluble at these higher temperatures. For a sample that has been through a heat/cool cycle, the peak integral returns to that found before heating, implying a very similar solubility and exchange rate between the worm‐like micelles and free molecule. In combination, these data correlate with our model above; the heat/cool cycle leads to subtle changes in packing as opposed to the formation of a new structure.

The heat/cool cycle results in interesting effects. For example, solutions of 2NapFF were loaded into a syringe either without or with a heat/cool cycle. After 18 h, the solutions behave very differently on being pushed out of the syringe (Figure 3 b; see video in the Supporting Information). The solution without a heat/cool cycle flows as expected for a slightly viscous solution. The solution that has been exposed to a heat/cool cycle can be pushed out as a single strand essentially. Solutions of 2NapFF at high pH can be gelled by adding a divalent cation.6a, 9 A homogeneous gel is formed after standing overnight (initially, local gelation occurs where the salt solution contacts the 2NapFF solution). As described previously,2a addition of a solution of CaCl_2_ to an as‐prepared solution of the gelator leads to formation of a turbid gel. A significantly more transparent gel is formed if the solution of CaCl_2_ is added to a solution removed immediately after the heating step, or to a pre‐heated solution that has been allowed to cool and rest at room temperature (Figure 4 a).

**Figure 4:**
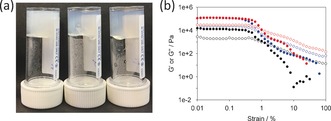
a) Gels formed by adding a solution of CaCl_2_ to a solution of 2NapFF (10 mg mL^−1^; pH 11). From left to right: gels formed from a solution as‐prepared, from a heated solution with the CaCl_2_ added just after heating, and a heated solution with the CaCl_2_ added after cooling. b) Strain sweeps for gels formed by adding CaCl_2_ to an as‐prepared solution of 2NapFF (10 mg mL^−1^; pH 11, (•○)), from a heated solution with the CaCl_2_ added just after heating (•○), and a heated and cooled solution with the CaCl_2_ added after cooling (•○). Key: G′ (filled symbols), G′′ (empty symbols).

The closest analogy we can find for the behavior that we observe here is reported by Stupp et al. with respect to peptide amphiphiles,^16^ where heating and cooling led to a change in the properties of the solution and greater alignment of structures. The heating and cooling was found to be necessary to allow “noodling”, for example. Their data suggested that the local packing was not changed by heating and cooling, but that the aggregates were dehydrated by heating, leading to filaments with significantly greater diameters. This increase in diameter was backed up by changes in the SAXS data.^16^ Hence, our systems are behaving differently.

Gels formed from the pre‐heated solutions behaved as described previously, with a *G*′ of 18.9±3.4 kPa, and a tan δ (*G*′′/*G*′) of 0.17±0.02 (Figure 4 b). Interestingly, the gels formed when the solution of the CaCl_2_ was added, either immediately after heating or after a cooling period, were significantly stiffer and very similar to one other. *G*′ was 119.4±1.4 kPa with a tan δ of 0.20±0.01 for the gels formed by adding CaCl_2_ immediately, and *G*′ was 122.7±4.1 kPa with a tan δ of 0.22±0.01 for the gels formed by adding CaCl_2_ after cooling (Figure 4 b). These are some of the highest reported values for such a gel. Cryo‐TEM of the gels shows that lateral association of the fibers occurs on addition of the calcium salt (Supporting Information, Figures S13–15).

This behavior is not restricted to 2NapFF. A number of other LMWGs show similar results (Supporting Information, Figures S16–S18). A prerequisite seems to be that the solutions form viscous solutions at high pH, implying that worm‐like micelles formed from this class of molecule behave in this manner. As we have described previously,6b these are the more hydrophobic examples. Less hydrophobic LMWGs form solutions that are not viscous and show no tendency to either become self‐supporting or to become more viscous (Supporting Information, Figures S19–S21).

In summary, we have shown that a simple heat/cool cycle results in a significant change in the physical properties of a solution of a LMWG. The heat/cool cycle results in an increase in the length of the worm‐like micelles and allows interesting new materials to be prepared. The rheological properties of the gels formed from solutions before heating or after a heat‐cool cycle are very different. The increase in extensional viscosity, for example, potentially allows electrospinning to be carried out.^17^ As well as the opportunities that this offers, we highlight that this may have important implications for experiments carried out in labs with different operating temperatures.

## Conflict of interest

The authors declare no conflict of interest.

## Supporting information

As a service to our authors and readers, this journal provides supporting information supplied by the authors. Such materials are peer reviewed and may be re‐organized for online delivery, but are not copy‐edited or typeset. Technical support issues arising from supporting information (other than missing files) should be addressed to the authors.

Supplementary

Supplementary
