# Supplementary material for: Opening a Can of Worm(‐like Micelle)s: The Effect of Temperature of Solutions of Functionalized Dipeptides
Source: Angew Chem Int Ed Engl. 2017 Jul 28;56(35):10467–70. doi: 10.1002/anie.201705604 (PMC5577516; doi:10.1002/anie.201705604)
Supplement: Supplementary file 2 — Supplementary [file ANIE-56-10467-s002.pdf]

Angewandte Chemie
Eine Zeitschrift der Gesellschaft Deutscher Chemiker

Supporting Information

Opening a Can of Worm(-like Micelle)s: The Effect of Temperature of Solutions of Functionalized Dipeptides

*Emily R. Draper, Hao Su, Christopher Brasnett, Robert J. Poole, Sarah Rogers, Honggang Cui, Annela Seddon, and Dave J. Adams**

anie_201705604_sm_miscellaneous_information.pdf
anie_201705604_sm_IMG_0977.mov

SUPPLEMENTARY INFORMATION

Materials and Methods

2NapFF was synthesised as described previously.[1] Deionised water was used throughout. The stock solution of sodium hydroxide was prepared from pellets, purchased from Sigma Aldrich. The pH was adjusted with HCl (2 M), which was prepared from a 12 M concentrated solution purchased from Sigma Aldrich. Calcium chloride was purchased from Sigma Aldrich; a stock solution of 200 mg/mL was prepared in water.

Preparation of solutions of 2NapFF. Solutions were prepared by suspending 2NapFF (500 mg) in water (38 mL), followed by the addition of sodium hydroxide (12 mL of a 0.1M aqueous solution). The suspension was stirred overnight, resulting in dissolution of the 2NapFF and the formation of a slightly viscous solution. The pH at this point was typically 10.8 to 11.2. Different solutions behaved similarly.

Heating Methods. For the samples where quantitative measurements were to be carried out, solutions were heated in an oil bath fitted with a temperature probe to the required temperature. The temperature of the solution is quoted, not that of the oil bath. Solutions were generally heated for 30 minutes; longer heating times had no apparent effect on the results. The solutions were then removed from the oil bath and allowed to stand quiescently overnight before measurement (typically, the samples could be inverted after around 1 hour).

For quick tests of inversion, the samples were heated with a hot air gun for a short period of time. They were then allowed to stand at room temperature.

Preparation of Gels. Gels were prepared by the addition of 66 μL of the stock solution of calcium chloride to 2 mL of a solution of 2NapFF. No mixing was carried out post addition, so local gelation occurred immediately around the drop. Over the period of several hours, the samples homogenized to give rheologically reproducible gels. The rheology of the gels was measured 18 hours after addition of the calcium salt. Three different samples were prepared: (i) the $CaCl_2$ was added to a fresh solution of 2NapFF; (ii) the $CaCl_2$ was added to a solution of 2NapFF that had just been heated to 40 °C for 30 minutes; the $CaCl_2$ was added to a solution of 2NapFF that had been heated to 40 °C for 30 minutes and then allowed to cool quiescently on the bench for 1 hour.

pH Measurement. A FC200 pH probe (HANNA instruments) with a 6 mm x 10 mm conical tip was used for pH measurements. The stated accuracy of the pH measurements is ±0.1.

Apparent pK_a Titration. To determine the apparent pK_a of each solution, a titration was performed by measuring the pH of the solutions and slowly lowering the pH by adding 5 µL aliquots of 0.1 HCl. The pH was then allowed to equilibrate before being

recorded. This was typically 3-5 minutes after the addition of the HCl. The temperature was maintained at 25 °C during the titration using a circulating water bath. To prevent a gel forming, the solutions were gently stirred using a stirrer bar, so keeping the sample liquid during the “titration” process.

Rotational Viscosity. A cone and plate system (50 mm cone) was used to measure the viscosities. 2 mL of a solution was transferred onto the plate for measurement by pouring. The viscosity of each solution was recorded under the rotation shear rate varying from 1 to 1000 s^{-1}. All the experiments were conducted at 25 °C. To minimise evaporation, the 2 mL of solution is significantly more than was needed to be covered by the plate. For the measurement of the effect of heating and cooling, the sample was heated to 40 °C for 2 minutes, and the viscosity measured. During this time, the amount of evaporation was minimal as observed visually from the amount of solution remaining on the plate. The sample was then cooled to 25 °C, and the viscosity re-measured after an equilibration period of 2 minutes. Again, visually minimal evaporation had occurred.

Small Amplitude Oscillatory Shear Rheology. Dynamic rheological measurements were performed using an Anton Paar Physica MCR301 rheometer using a vane and cup measuring system. The gels were prepared in 7 mL Sterlin vials and loaded straight into the rheometer after 18 hours. All experiments were performed at 25 °C. Strain sweeps were performed from 0.1 % to 1000 % at a frequency of 10 rad/s. Frequency sweeps were performed from 1 to 100 rad/s at a strain of 0.5 % (all the gels were in the linear viscoelastic (LVE) region at this strain).

Extensional Viscosity. The CaBER comprises two circular stainless steel plattens with a diameter of 4 mm with an initial separation of ~2 mm. A small sample of each solution was loaded between the plattens using a syringe (without a needle to minimise the shear) to form a cylindrical sample. A rapid axial step strain is imposed (~50 ms) until a final height (~ 9 mm) is reached and an unstable filament is formed. Subsequently, the sample filament breaks up under the combined action of capillary and extensional viscoelastic forces.[2]

Small angle X-ray scattering (SAXS). All X-ray scattering was performed on a SAXSLAB Ganesha 300XL instrument in a Q range of 0.006-0.26$Å^{-1}$, with an exposure time of 7200 seconds per sample. The solution of 2NapFF in water was transferred directly to an X-ray capillary for measurement. For investigating the properties of 2NapFF after a heat/cool cycle, the solution was heated at 70 ^{o}C for 1 hour before being transferred to an X-ray capillary for measurement, sealed, and left to cool for a period of 24 hours. Additional measurements on an empty capillary and a capillary filled with water were taken to account for tube width corrections.

Small angle neutron scattering (SANS). The measurements were performed using the SANS2D instrument (STFC ISIS Pulsed Neutron Source, Oxfordshire, UK). A collimation length of 12m and incident wavelength (λ) range of 1.75 to 12.5 Å was employed. Data were measured simultaneously on two 1 m^2 detectors to give a Q-range [$Q = 4\pi\sin(\theta/2)/\lambda$, where θ is the scattering angle] of 0.0015 to 0.5 $Å^{-1}$. The small-angle detector was positioned 12m from the sample and offset vertically 60 mm and sideways 100 mm. The wide-angle detector was positioned 5 m from the sample, offset sideways by 860 mm and rotated to face the sample. The beam

diameter was 8 mm and the samples were placed in 2 mm quartz cuvettes and measured for ~60 minutes. The scattering data were normalized for the sample transmission and background corrected D_2O and corrected for the linearity and efficiency of the detector response using the Mantid framework.[3] The scattering data were then fitted in the SasView software (version 3.1.1)[4] to a hollow cylinder model.

Cryo-TEM. Cryogenic TEM imaging was performed on the FEI Tecnai 12 TWIN Transmission Electron Microscope, operating at 100 kV. The solutions were diluted five times with water to reduce their viscosity and 6 µL of sample solution was placed on a holey carbon film supported on a TEM copper grid (Electron Microscopy Services, Hatfield, PA). The gels were similarly diluted, although they were very rigid and therefore this process was difficult. All the TEM grids used for cryo-TEM imaging were treated with plasma air to render the lacey carbon film hydrophilic. A thin film of the sample solution was produced using the Vitrobot with a controlled humidity chamber (FEI). After loading of the sample solution, the lacey carbon grid was blotted using preset parameters and plunged instantly into a liquid ethane reservoir precooled by liquid nitrogen. The vitrified samples were then transferred to a cryo-holder and cryo-transfer stage which was cooled by liquid nitrogen. To prevent sublimation of vitreous water, the cryo-holder temperature was maintained below -170 °C during the imaging process. All images were recorded by a SIS Megaview III wide-angle CCD camera.

Image Analysis. For cryo-TEM, fibre width measurements were carried out using ImageJ. The scale bar was used to set the scale for the width measurement. Between 78 and 80 measurements of fibres were used to create the histograms. These measurements were done on several images of the same gels to ensure the widths were representative.

Fluorescence Spectroscopy. Data were collected on a Cary Eclipse Fluorescence Spectrophotometer (Agilent Technologies). An excitation wavelength of 265 nm was used, with slit widths of 5 nm. 1 cm path length cuvettes were used.

Infra-Red Spectroscopy. Data were collected on a using an ATR crystal on a Cary 630 FTIR (Agilent Technologies). A background of NaOD in D_2O was used, with the samples prepared also in D_2O.

NMR Spectroscopy. ^{1}H NMR spectra were recorded on a Bruker Avance III 400 MHz spectrometer. Solutions were prepared as above, replacing the NaOH and H2O with NaOD and D2O respectively. Ethanol was added as an internal standard.

Supplementary Figures and Tables

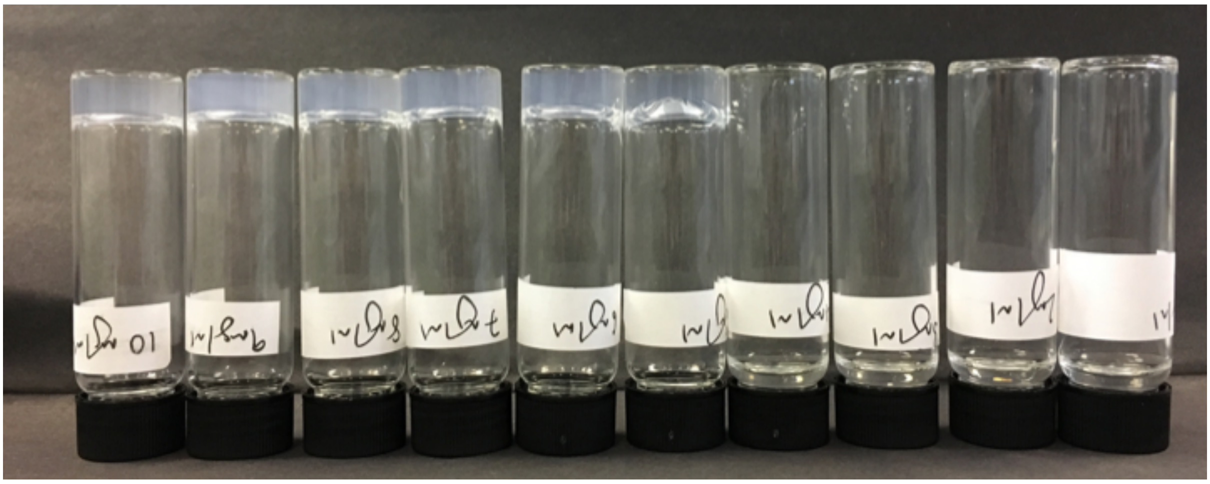

Figure S1. Photographs of solutions of 2NapFF at pH 10.97 at different concentrations after a heat-cool cycle and resting for 4 hours. From left to right, the concentration decreases from 10 mg/mL to 1 mg/mL. The solutions from 10 mg/mL to 6 mg/mL could be inverted easily. The solution at 5 mg/mL was weaker than the others, as can be seen from the photo, where some flow had begun. The solutions at 3 and 4 mg/mL were visibly more viscous after standing overnight, but still could not be inverted.

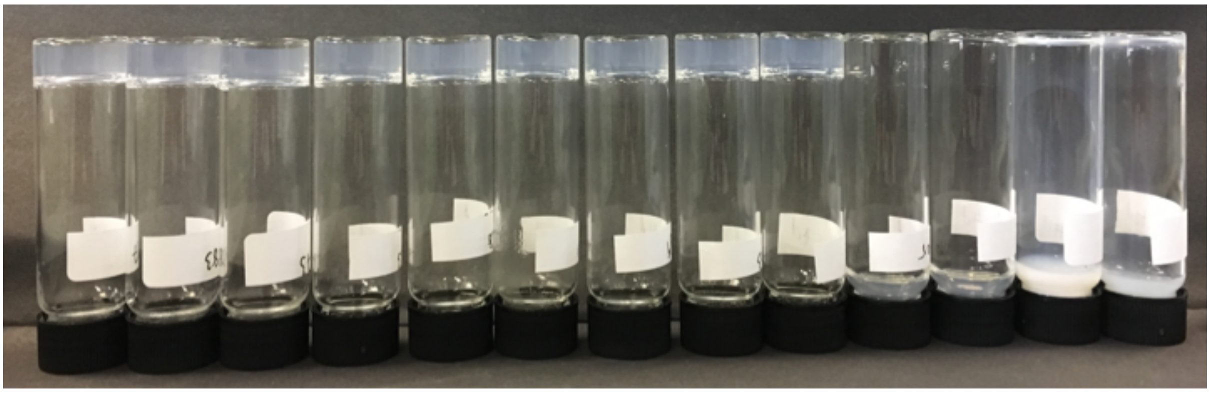

Figure S2. Photographs of solutions of 2NapFF at a concentration of 10 mg/mL at different pH after a heat-cool cycle and resting for 4 hours. From left to right, the pH was 10.97, 10.83, 10.73, 10.53, 10.28, 10.00, 9.89, 9.53, 9.48, 9.05, 8.46, 7.26, and 6.59. The solutions at pH 7.26 and pH 6.59 are cloudy as they are close to the pK_a of 2NapFF and hence the solubility of the 2NapFF is low.

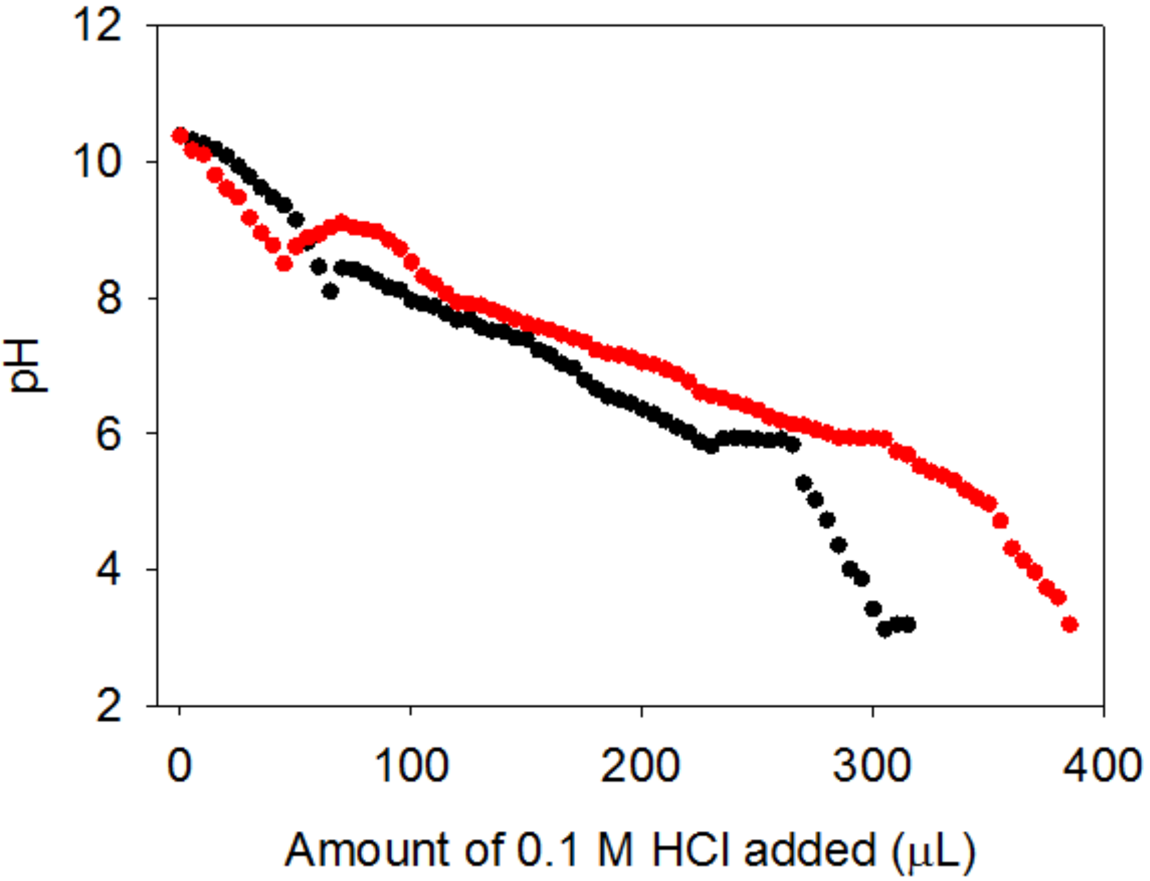

Figure S3. Apparent pK_a titration of a solution of 2NapFF at a concentration of 10 mg/mL before heating (black data) and after a heat-cool cycle (red data). All data were collected at 25 °C.

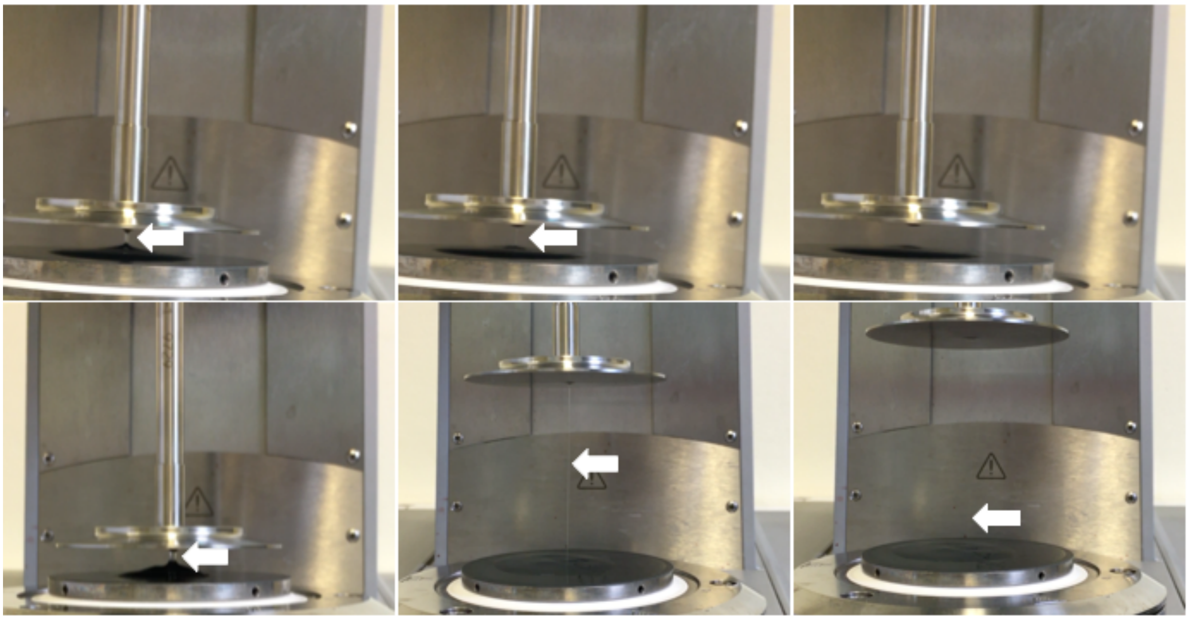

Figure S4. Photographs of solutions of 2NapFF on the rheometer as the top plate is raised after a viscosity measurement. The top images show a solution with no heat-cool cycle. Some extensional viscosity is observed (the white arrow highlights this), but the filament quickly breaks. The middle image shows the filament just before breaking, and the right image just after breaking. The bottom images are for a sample that had been through a heat-cool cycle. The filament exists for a much greater distance. The filament broke just after the point shown on the right image. The top plate on the rheometer is being continuously raised throughout, so these data are purely qualitative.

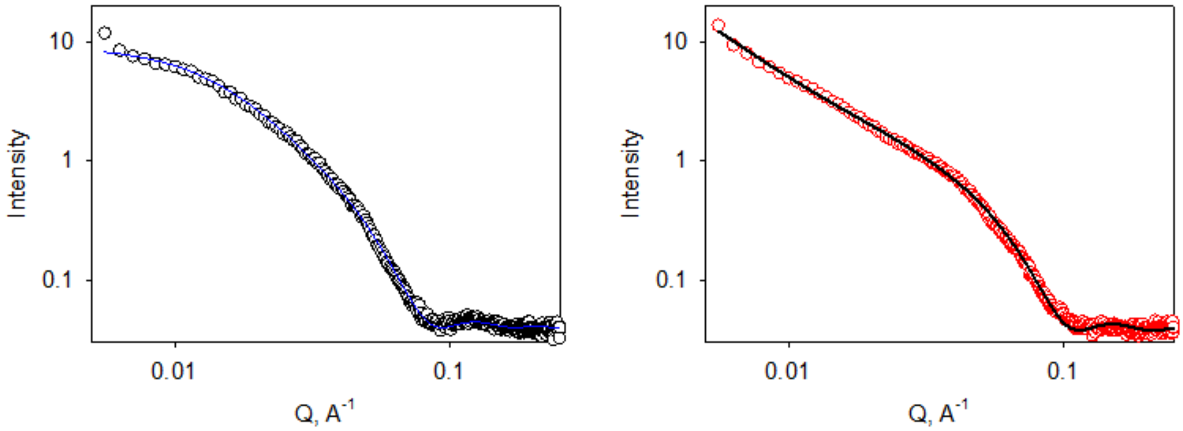

Figure S5. SAXS data and fits. (Left) shows the data (open circles) and fit (line) for a solution of 2NapFF at a concentration of 10 mg/mL. (Right) shows the data and fit for the same solution after a heat/cool cycle. The fits are to a flexible cylinder with the parameters shown below in Table S1.

	Pre-heating	After heat/cool
Background / cm^{-1}	0.0398 ± 0.00013	0.037911 ± 0.000128
Kuhn Length / Å	63.0 ± 2.4	339.7 ± 3.4
Length / Å	949.6 ± 5.6	4976.4 ± 232.8
Radius / Å	40.5 ± 0.2	34.0 ± 0.1
Scale	0.0059 ± 1.1e-5	0.01058 ± 1.99e-5
χ^2	4.1614	3.7879

Table S1. The model fit parameters generated by fitting the flexible cylinder models to the SAXS data in SasView.

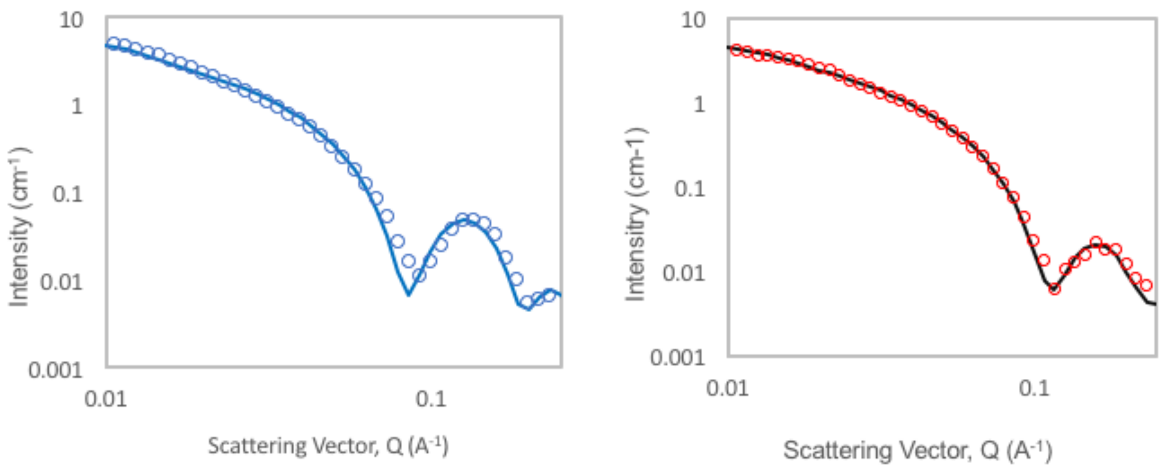

Figure S6. SANS data and fits. (Left) shows the data (open circles) and fit (line) for a solution of 2NapFF at a concentration of 10 mg/mL. (Right) shows the data and fit for the same solution after a heat/cool cycle. The fits are to a hollow cylinder with the parameters shown below in Table S2.

	Pre-heating	After heat/cool
Background / cm^{-1}	0.0040 ± 0.00028	0.0040 ± 0.00028
Core Radius / Å	19.1 ± 0.2	9.2 ± 0.3
Radius / Å	36.9 ± 0.1	31.2 ± 0.1
Length / Å	401.1 ± 4.1	3127.9 ± 3.2
Scale	0.0046 ± 7.54e-5	0.0053 1 ± 6.48e-5
χ^2	17.818	11.788

Table S2. The model fit parameters generated by fitting the flexible cylinder models to the SANS data in SasView.

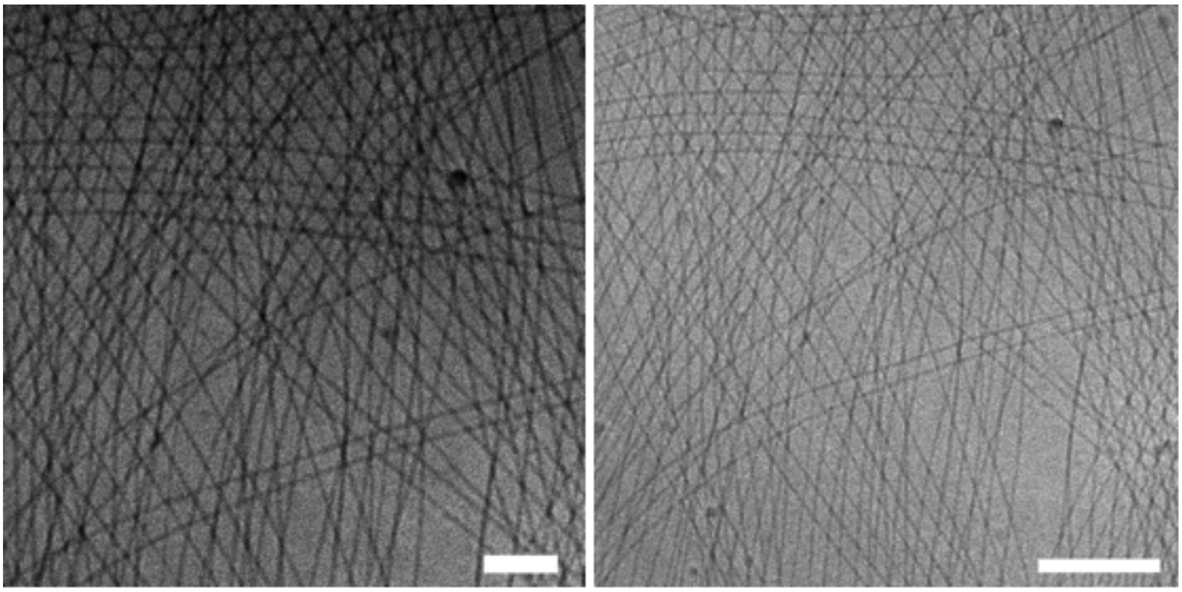

Figure S7. Cryo-TEM of a solution of 2NapFF as prepared. The scale bars represent 200 nm in both cases.

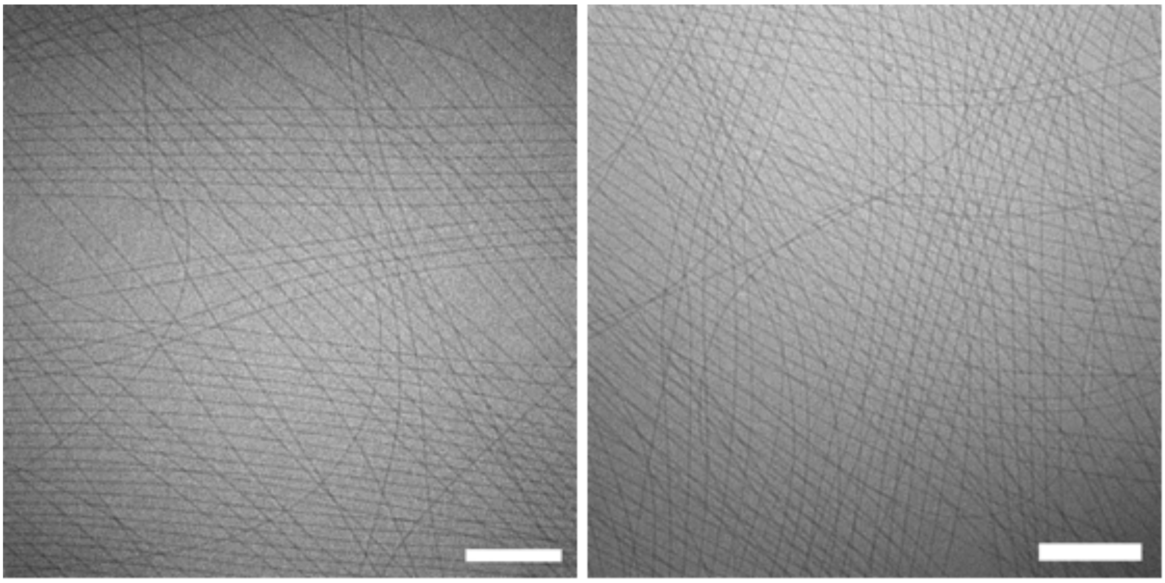

Figure S8. Cryo-TEM of a solution of 2NapFF after a heat-cool cycle. The scale bars represent 200 nm in both cases.

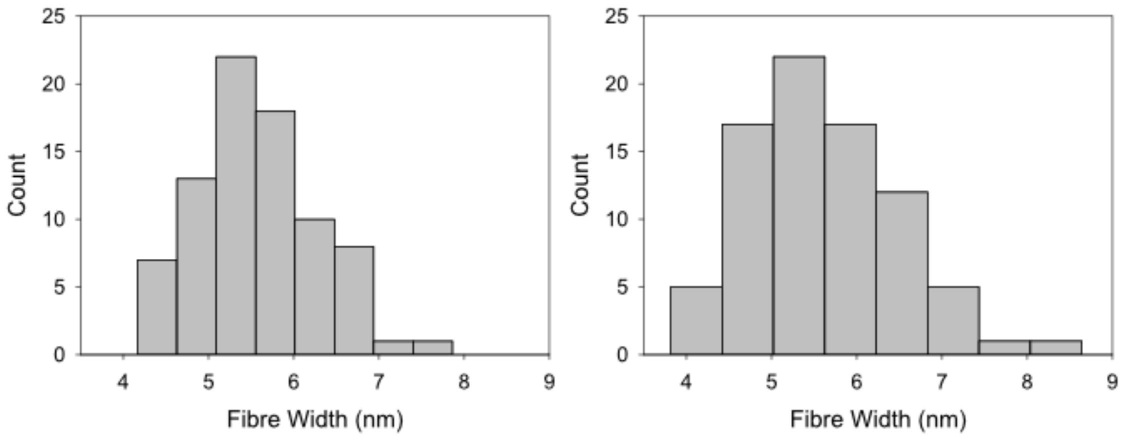

Figure S9. Histograms showing fibre widths as measured from the cryo-TEM data for a solution of 2NapFF before heating (left) and after a heat-cool cycle (right). The measured distributions are 5.6 ± 0.7 nm and 5.6 ± 0.9 nm respectively.

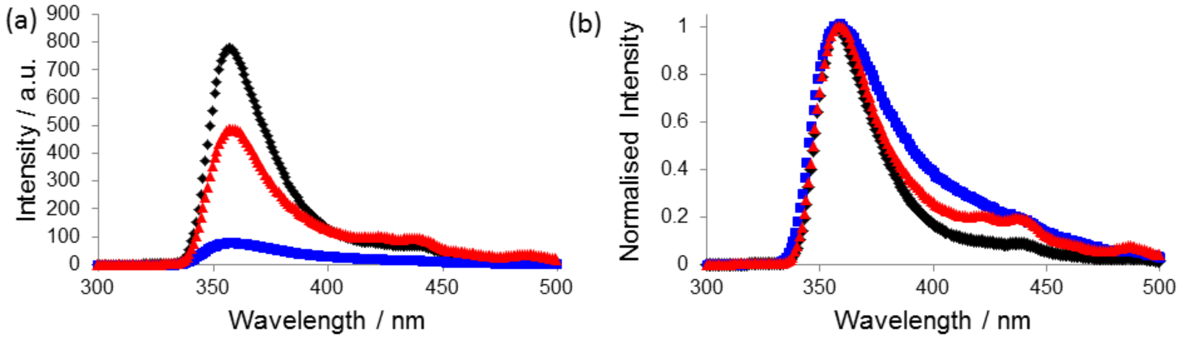

Figure S10. Fluorescence spectra (excitation wavelength 265 nm) for a solution of 2NapFF (10 mg/mL, pH 10.5) before heating (black), immediately after heating (blue), and after standing overnight at room temperature following heating (red). (a) shows the absolute intensities measured. (b) shows the data in (a) normalized to the intensity at 355 nm.

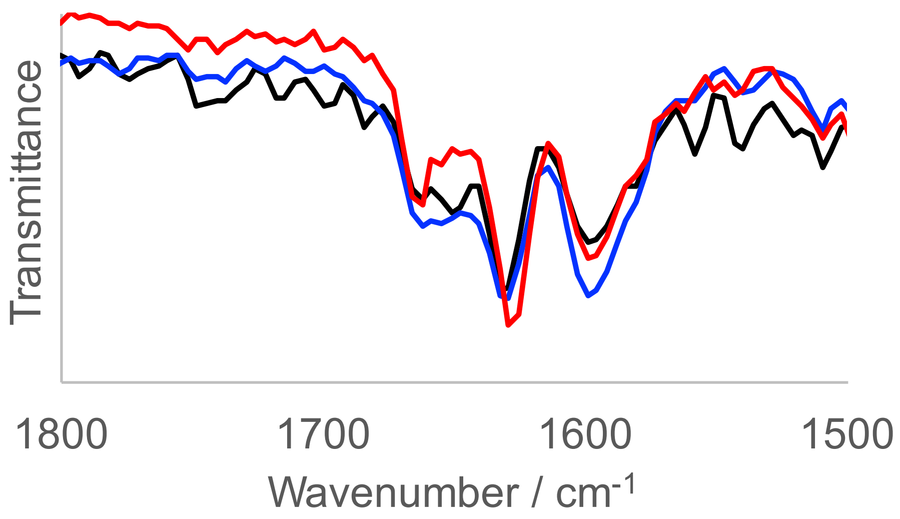

Figure S11. Infra-red data for a solution of 2NapFF (10 mg/mL, pH 10.5) before heating (black), immediately after heating (blue), and after standing overnight at room temperature following heating (red). The data are shown for solutions in D_2O. A solution of NaOD in D_2O was used as the background.

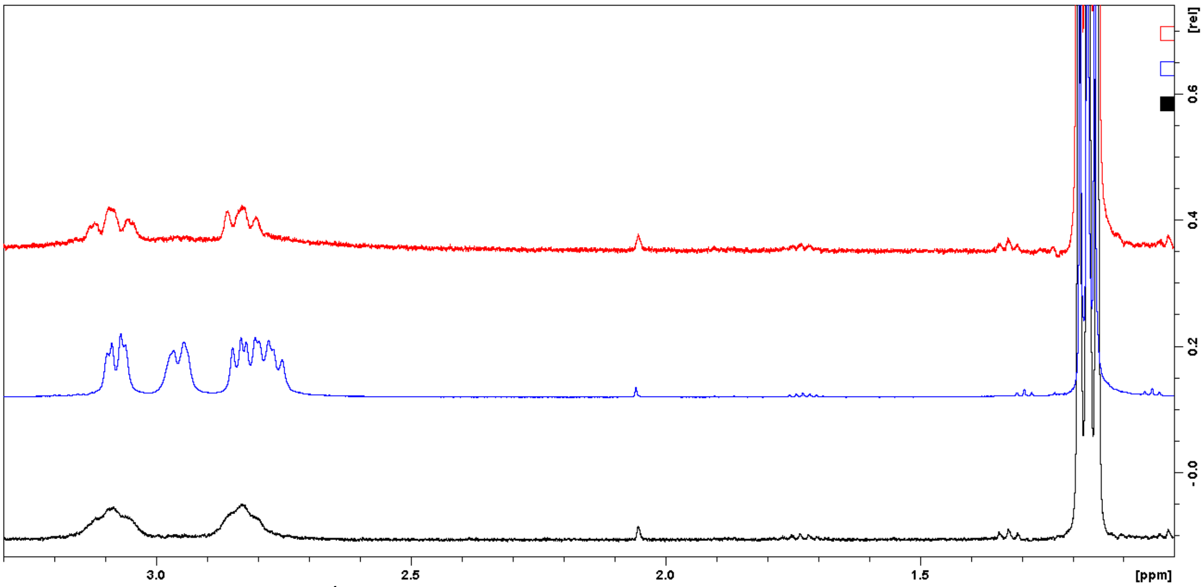

Figure S12. A partial ^{1}H NMR spectrum for a solution of 2NapFF (10 mg/mL, pH 10.5) in D_2O before heating (black), immediately after heating (blue), and after standing overnight at room temperature following heating (red). The peak at 1.16 ppm is the CH_3 group on ethanol added as an internal standard. The peaks between 2.8 and 3.2 ppm are the CH_2 protons on the phenylalanine groups. The relative integral before and after heating between the standard and the 2NapFF protons is almost identical (1:5.12 and 1:5.17 respectively). On heating, the solubility of the 2NapFF increases as can be seen by the sharpening of the peaks and the increase in integral of the 2NapFF (integral of ethanol:2NapFF = 1.765 at 40 °C).

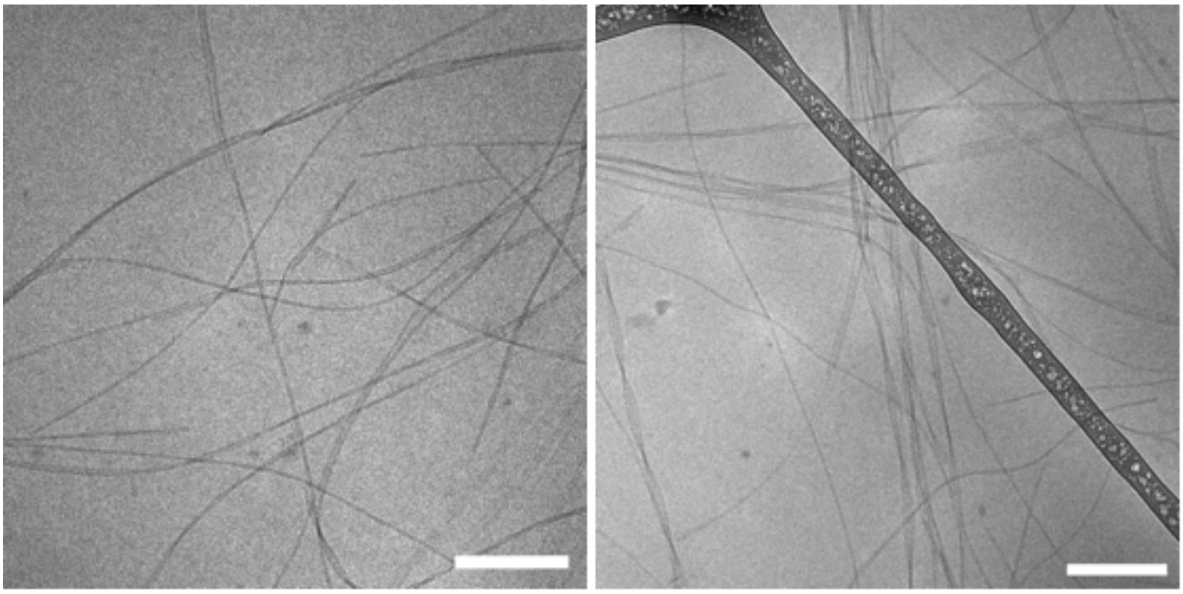

Figure S13. Cryo-TEM of a gel of 2NapFF as prepared by addition of $CaCl_2$. The scale bars represent 200 nm in both cases. Note the small circular structures are ice crystals and the darker, wider object in the right-hand image is the grid.

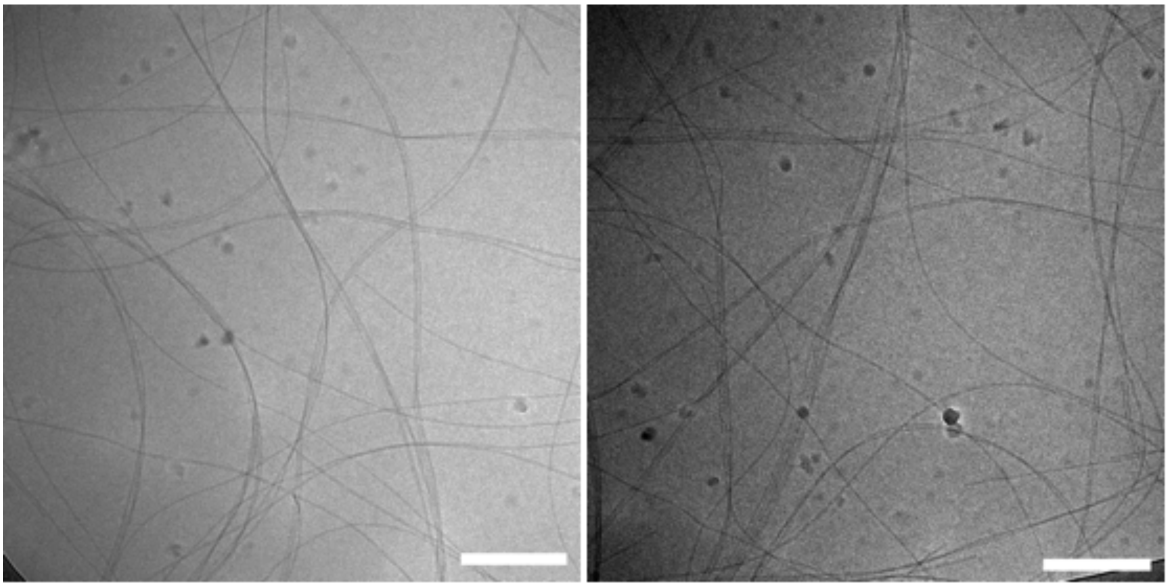

Figure S14. Cryo-TEM of a gel of 2NapFF where the $CaCl_2$ was added after a heat-cool cycle. The scale bars represent 200 nm in both cases. Note the small circular structures are ice crystals.

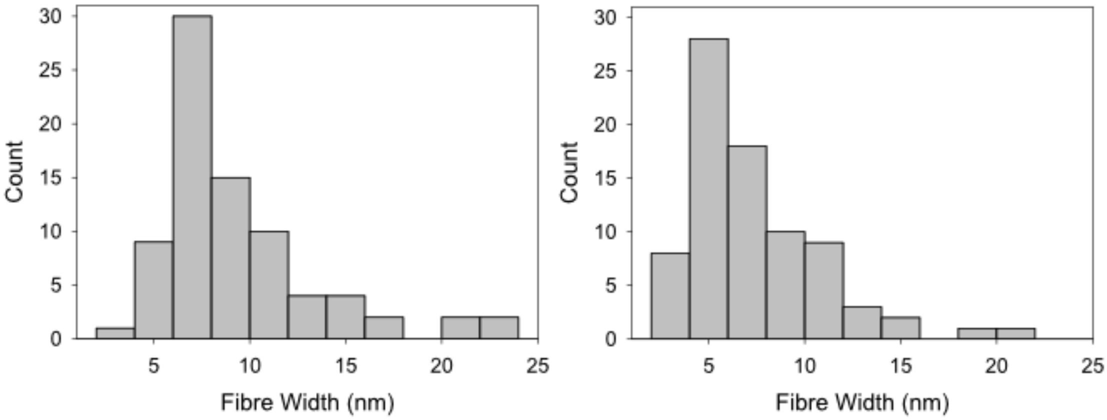

Figure S15. Histograms showing fibre widths as measured from the cryo-TEM data for gels of 2NapFF formed by adding the $CaCl_2$ before heating (left) and after a heat-cool cycle (right). The measured distributions are 9.5 ± 4.8 nm and 7.4 ± 3.5 nm respectively.

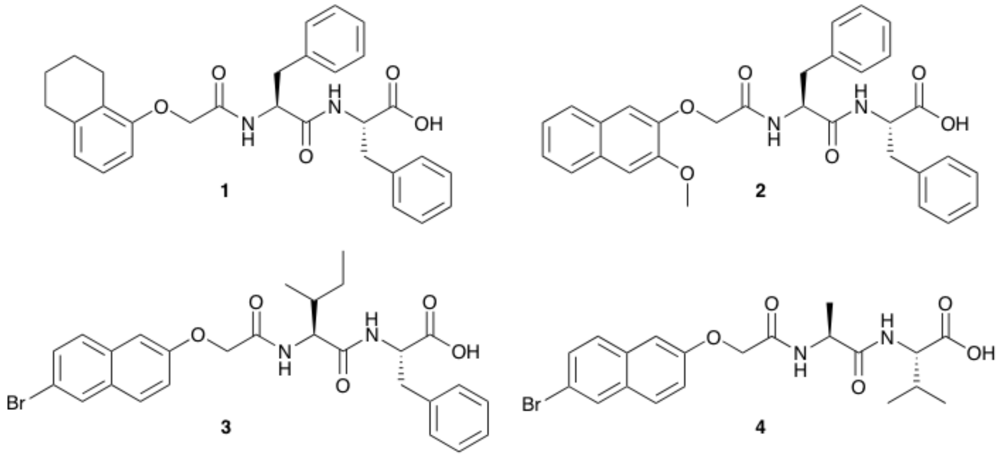

Figure S16. Chemical structures of LMWG for which solutions become more viscous after a heat-cool cycle. The LMWG were prepared as described previously.[1, 5] All solutions were tested at a concentration of 10 mg/mL of the dipeptide and a pH of 10.5.

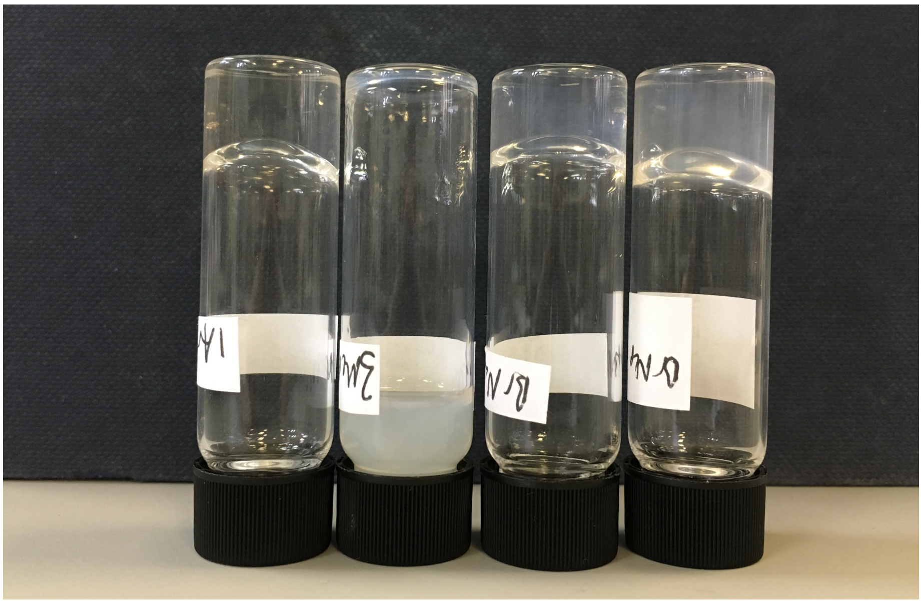

Figure S17. Photographs of (from left to right) LMWG **1**, **2**, **3**, and **4** (see Fig. S13 for structures) after a heat-cool cycle. All solutions were tested at a concentration of 10 mg/mL of the dipeptide and a pH of 10.5. Note, for **2**, the viscosity increase does not translate into the ability to invert the vial after standing overnight, but the samples can almost be inverted after around 1 hour; after this, the sample gradually becomes less able to be inverted and the turbidity increases. We interpret this as further aggregation of the structures over longer time periods. However, the viscosity is still significantly increased after standing overnight as compared to the initial solutions (see Fig. S15b).

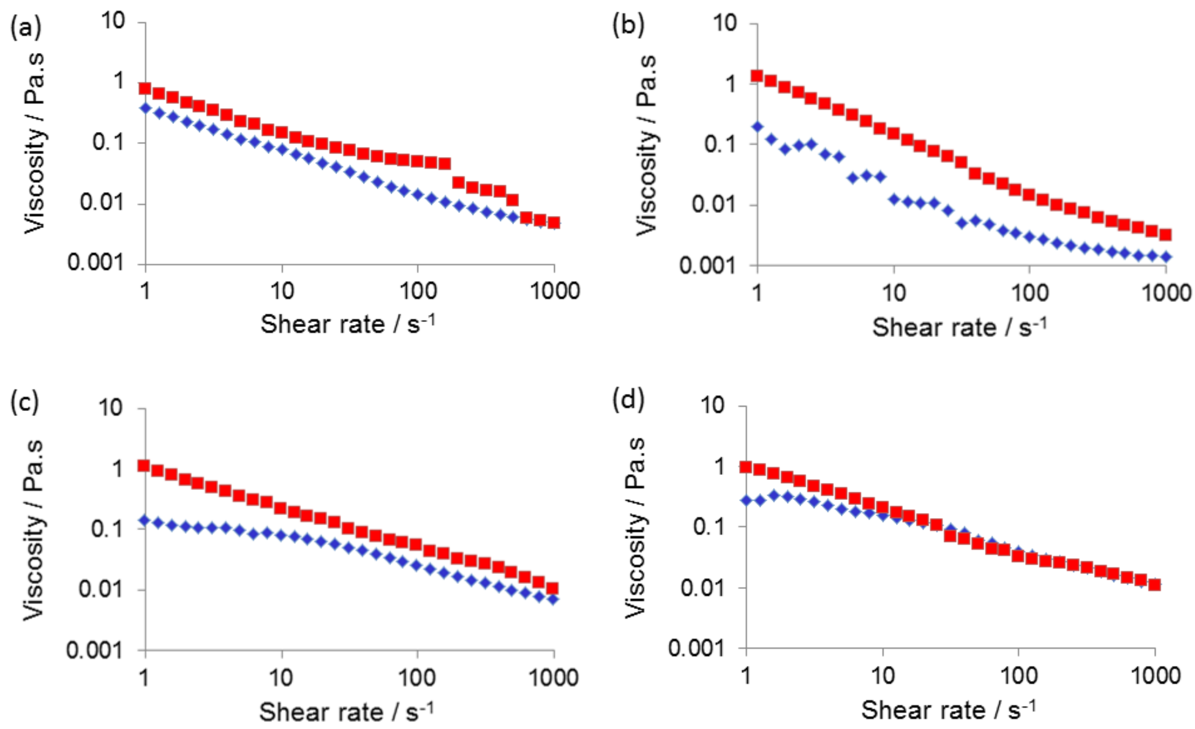

Figure S18. Viscosity of solutions of LMWG (10 mg/mL; pH 10.5) at 25 °C (blue data), overlaid with data for solutions that were heated to 40 °C and re-cooled to 25 °C and allowed to stand at 25 °C for 18 hours (red). The LMWG are (a) 1; (b) 2; (c) 3; (d), with the structures oft he LMWG shown in Fig. S13.

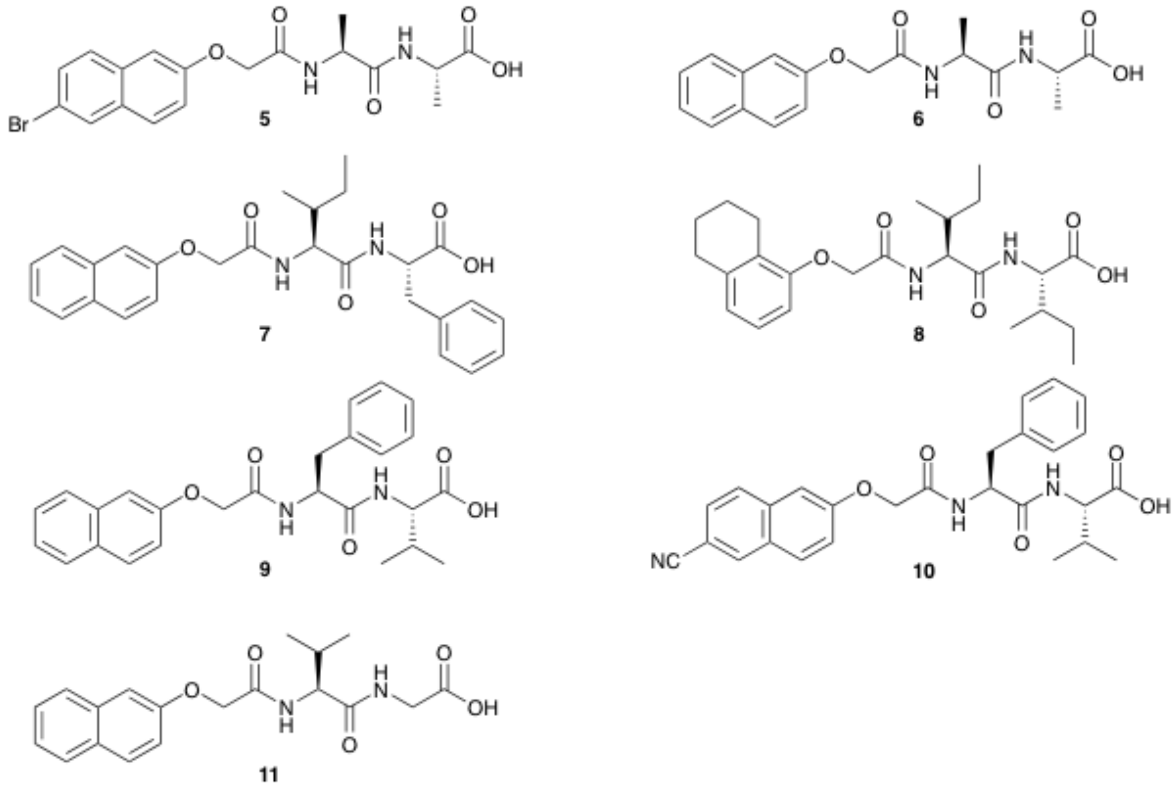

Figure S19. Chemical structures of LMWG where the solutions do not become more viscous after a heat-cool cycle. The LMWG were prepared as described previously.[1,

[5] All solutions were tested at a concentration of 10 mg/mL of the dipeptide and a pH of 10.5.

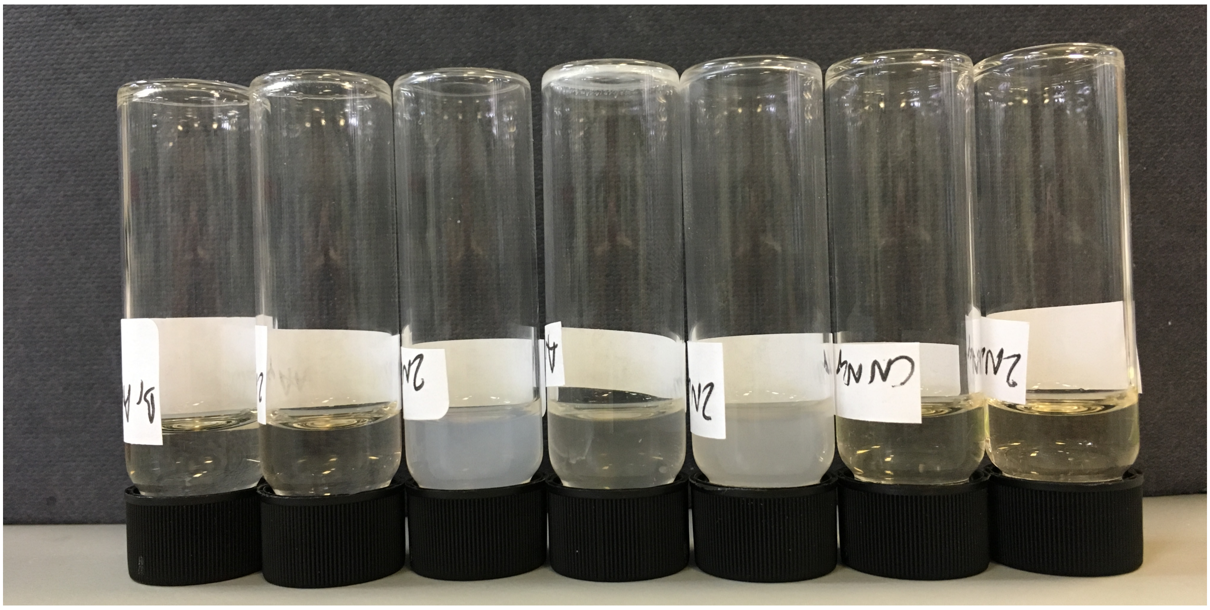

Figure S20. Photographs of (from left to right) LMWG **5**, **6**, **7**, **8**, **9**, **10**, and **11** (see Fig. S16 for structures) after a heat-cool cycle. All solutions were tested at a concentration of 10 mg/mL of the dipeptide and a pH of 10.5.

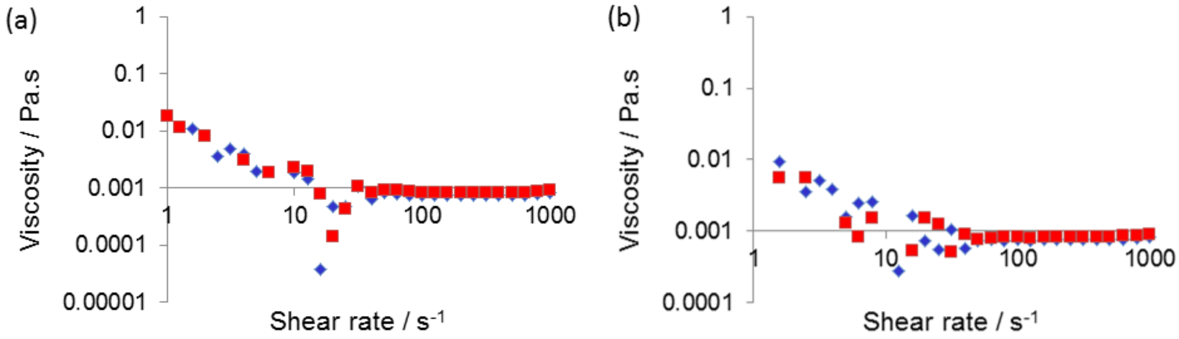

Figure S21. Example viscosity data from solutions of LMWG (10 mg/mL; pH 10.5) at 25 °C (blue data), overlaid with data for solutions that were heated to 40 °C and re-cooled to 25 °C and allowed to stand at 25 °C for 18 hours (red). The LMWG are (a) **8**; (b) **10**, with the structures of the LMWG shown in Fig. S16.

References

[1] L. Chen, S. Revel, K. Morris, L. C. Serpell, D. J. Adams, *Langmuir* **2010**, *26*, 13466-13471.

[2] L. E. Rodd, T. P. Scott, J. J. Cooper-White, G. H. McKinley, *Applied Rheology* **2005**, *15*, 12-27.

[3] O. Arnold, J. C. Bilheux, J. M. Borreguero, A. Buts, S. I. Campbell, L. Chapon, M. Doucet, N. Draper, R. Ferraz Leal, M. A. Gigg, V. E. Lynch, A. Markvardsen, D. J. Mikkelson, R. L. Mikkelson, R. Miller, K. Palmen, P. Parker, G. Passos, T. G. Perring, P. F. Peterson, S. Ren, M. A. Reuter, A. T. Savici, J. W. Taylor, R. J. Taylor, R. Tolchenov, W. Zhou, J. Zikovsky, *Nuclear Instruments and Methods in Physics Research Section A: Accelerators, Spectrometers, Detectors and Associated Equipment* **2014**, *764*, 156-166.

[4] http://www.sasview.org/.
[5] J. K. Gupta, D. J. Adams, N. G. Berry, *Chemical Science* **2016**, *7*, 4713-4719.
